# Prognostic value of miR-21 in gliomas: comprehensive study based on meta-analysis and TCGA dataset validation

**DOI:** 10.1038/s41598-020-61155-3

**Published:** 2020-03-06

**Authors:** Guli Jiang, Jing Mu, Xing Liu, Xiangni Peng, Feiya Zhong, Wenliang Yuan, Fang Deng, Xiaoning Peng, Sihua Peng, Xiaomin Zeng

**Affiliations:** 1 Department of Epidemiology and Health Statistics, Xiangya Public Health School, Central South University, Changsha, 410078 Hunan P.R. China; 2 Department of Medicine, Guangzhou Cadre Sanatorium, Guangzhou, 510530 Guangdong P.R. China; 3 Department of Medicine, Xiang’an Hospital, Xiamen University, Xiamen, 361102 Fujian P.R. China; 4 Department of Mathematics, The Ohio State University, Columbus, 43210 OH USA; 5 Department of Genetics, College of Life Science, Wuhan University, Wuhan, 430072 Hubei P.R. China; 6 Department of Developmental Biology, School of Fisheries and Life Science, Shanghai Ocean University, Shanghai, 201306 Shanghai P.R. China; 7 Department of Pathology and Pathophysiology, School of Medicine, Hunan Normal University, Changsha, 410013 Hunan P.R. China; 8 Department of Pathophysiology, School of Medicine, Jishou University, Jishou, 416000 Hunan P.R. China

**Keywords:** CNS cancer, CNS cancer

## Abstract

Recent studies have highlighted the value of microRNA-21 (miR-21) as a prognostic biomarker in gliomas. However, the role of miR-21 in predicting prognosis remains controversial. We performed a comprehensive study based upon a meta-analysis and The Cancer Genome Atlas (TCGA) glioma dataset validation to clarify the prognostic significance of miR-21 in glioma patients. In this study, we searched Embase, PubMed, Web of science, CNKI, SinoMed, and Wanfang databases for records up to May 2018. Relevant data were extracted to assess the correlation between miR-21 expression and survival in glioma patients. Pooled hazard ratios (HRs) with 95% confidence intervals (CIs) were used to describe association strength. We further used multivariate Cox regression analysis to assess miR-21 expression in the TCGA glioma dataset to validate the relationship between miR-21 expression and survival. Nine studies were included in the meta-analysis. Among them, eight studies provided data on overall survival (OS) with a pooled HR of 1.91 (95% CI: 1.34, 2.73), indicating that higher expression of miR-21 was significantly associated with worse OS in glioma patients; for the other study, which provided data on progression-free survival (PFS), no statistically significant HR was reported for PFS in the glioma patients (HR = 1.23, 95% CI: 0.41, 3.72). A multivariate Cox regression analysis of the miR-21 expression in the TCGA glioma dataset revealed that overexpression of miR-21 was a potential independent prognostic biomarker of poorer OS (HR = 1.27, 95% CI: 1.01, 1.59) and poorer PFS (HR = 1.46, 95% CI: 1.17, 1.82). Our findings suggest that higher expression of miR-21 is correlated with poorer glioma prognosis.

## Introduction

Gliomas are one of the most common central nervous system (CNS) glial neoplasms, accounting for 30% of all CNS tumors and 80% of malignant brain tumors^1^, respectively. According to the World Health Organization (WHO) classification, gliomas are classified as grade I through grade IV^2^. Low-grade gliomas (LGG, grades I–II) are well-differentiated and grow slowly, while high-grade gliomas (HGG, grades III–IV) are characterized by poor differentiation and rapid progression, and the prognosis of HGG is usually poor. Grade IV glioma, known as glioblastoma multiforme (GBM), is extremely aggressive, and comprises 55.1% of all gliomas^3^. Although recent progress has been made in surgical and radiotherapy techniques, the prognoses of GBM patients have not significantly improved^4,5^, and GBM remains one of the most incurable cancers^6^, with a 5-year survival rate of only 5.1%^3^. Some clinical factors impact the prognosis of glioma patients, such as age at diagnosis, WHO grade, duration of symptoms, tumor size, postoperative treatment, and karnofsky performance score (KPS)^7,8^. However, studies have shown that biological alterations in specific genes or molecules can affect the prognosis of glioma patients^7,8^. Therefore, clinical factors alone are not sufficient to evaluate the prognoses of glioma patients, and further research is needed to search for better prognostic biomarkers.

Micro-RNAs (miRNAs) are a class of small non-coding RNAs of 18–25 nucleotides with the ability to regulate gene expression at a post-transcriptional level by targeting messenger RNA (mRNA);^9,10^ miRNAs serve as key regulatory components in various biological processes, including cell proliferation, differentiation, angiogenesis, and apoptosis^11,12^. Dysregulation of miRNAs may lead to certain pathological states, such as cancer^13^. Recently, many studies have revealed that miRNAs can function as oncogenes or tumor suppressors in cancer, affecting the clinical outcome^14,15^. Studies have shown that miRNA expression is related to prognosis in patients with gliomas; for example, elevated expressions of microRNA-21 (miR-21), microRNA-10b (miR-10b), and microRNA-221/222 (miR-221/222) in patients with gliomas are correlated with shorter overall survival (OS) time^16–18^. This suggests that for the patients with gliomas, survival outcome can be predicted; therefore, miRNA expression may be clinically useful for management and prognosis.

Overexpressions of miR-21, also known as oncomiR, has been reported in various cancers, including gliomas^19–22^. Previous studies have shown that overexpression of miR-21 is associated with poor survival of glioma patients^23–29^. However, two studies reported insignificant correlations between miR-21 and prognosis in glioma patients^30,31^.

Meta-analysis is a method that allows quantitative analysis of the results of multiple independent studies, and has been used extensively to analyze the relationship between specific genes and prognosis in cancer patients^32–34^. Because of the inconsistent findings on the prognostic value of miR-21 in gliomas, a literature-based meta-analysis would be beneficial. A meta-analysis concerning the association between miR-21 expression and OS in glioma patients was published in 2016;^35^ however, several new studies on miR-21 expression and OS in glioma patients have been published since then. The Cancer Genome Atlas (TCGA) dataset has a large sample size of glioma patients, and the data have been standardized. Therefore, we performed an updated literature-based meta-analysis and also analyzed the miR-21 levels obtained from the TCGA database (https://portal.gdc.cancer.gov/) to elucidate the prognostic significance of miR-21 expression in glioma patients.

## Results

### Meta-analysis. study selection

**Figure 1:**
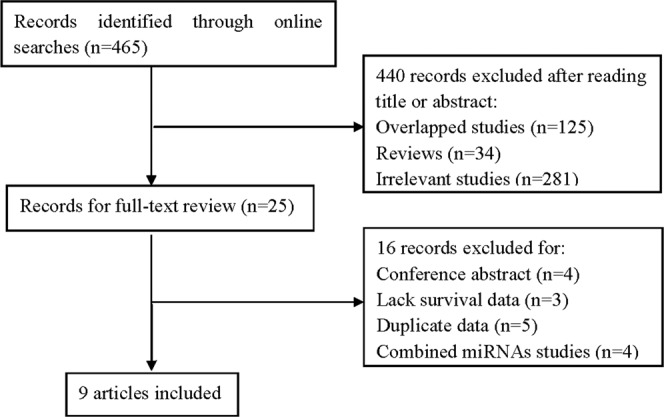
Flow chart of the study selection process.

A total of 465 articles were identified from the literature databases according to the inclusion criteria. Of these articles, 440 articles were removed because they were reviews, or were overlapped and irrelevant studies. Of the remaining 25 candidates, 16 articles were excluded because of one of the following: conference abstract (n = 4), lacking sufficient data to calculate hazard ratios (HRs) (n = 3), duplicated data (n = 5), and studying a set of microRNAs but not miR-21 alone (n = 4). Finally, nine articles were enrolled in our evaluation of the association between miR-21 expression and glioma prognosis. A flow chart of the eligible study selection process is shown in Fig. 1.

### Characteristics of enrolled studies

The nine included studies for this meta-analysis were cohort studies, and the main characteristics of these nine studies are summarized in Table 1. The enrolled nine studies from China (n = 5), USA (n = 2), Denmark (n = 1), and Austria (n = 1) were published between 2010 and 2017, and included a total of 1,059 glioma patients. Of the nine studies, the tumor grades of the glioma patients were heterogeneous. Three studies included patients with grades I–IV, two studies with grades II–IV, one study with grades III–IV, and three studies with grade IV only. The expressions of miR-21 were all examined in glioma tissues. The miR-21 expressions were verified by quantitative real-time polymerase chain reaction (qRT-PCR) in eight studies, and by *in situ* hybridization (ISH) in one study. The follow-up periods of the nine studies ranged from 44 months–120 months. The cut-off values used to define the high- or low-expression of miR-21, the adopted therapeutic regimen for the glioma patients, and the reference of miR-21 quantified in the tumor tissues in the nine included studies were different. Among the nine included studies, one study^31^ provided data on progression-free survival (PFS), and the other eight studied OS. The values of HR and 95% confidence interval (95% CI) were extracted from original data in six studies, and in the other three studies, the values were estimated using Kaplan-Meier curves (K-M curves) (n = 2) or original data (n = 1). A multivariate Cox regression analysis was performed in five studies^23–26,29^, and the adjustment variables were age, sex, KPS and the malignancy grade of glioma, among others. The Newcastle-Ottawa scales (NOSs) for all the eligible studies were assigned more than five stars, indicating a high methodological quality in the included studies.

**Table 1:** Characteristics of the enrolled studies.

First Author, Publication Year	Location of Sample Collection		n	miRNA Source	miRNA Assay	plain housekeeping miRNAs	Cut-off*(Actual Value)	Grade	Follow-up (months)	Outcome	HR (95% CI)	Extracting Method	Adjustment Variables	NOS
Zhang^28^	China	(Asian)	92	tissue	qRT-PCR	U6	Mean (—)	III-IV	96	OS	3.401 (1.296, 8.922)	Reported	—	7
Qu^27^	China	(Asian)	35	tissue	qRT-PCR	RNU6B	1.5-fold (—)	II-IV	72	OS	2.66 (1.02, 6.92)	K-M curve	—	5
Shi^23^	China	(Asian)	198	tissue	qRT-PCR	GAPDH	median (—)	II-IV	102	OS	1.634 (1.083, 2.467)	Reported	Age, Sex, KPS, WHO grade	7
Sathyan^30^	USA	(Non-Asian)	69	tissue	qRT-PCR	EEF1A	median (—)	IV	90	OS	1.63 (0.82, 3.22)	K-M curve	—	5
Barbano^26^	USA	(Non-Asian)	185	tissue	qRT-PCR	RNU48	mean (—)	IV	120	OS	1.19 (1.01, 1.41)	Reported	MGMT unmethylated, IDH1 mutation, Treatment, Recurrence	7
Hermansen^29^	Denmark	(Non-Asian)	189	tissue	ISH	U6	mean (—)	I-IV	70	OS	1.545 (1.002, 2.381)	Reported	Age, WHO grade	9
Wu^24^	China	(Asian)	152	tissue	qRT-PCR	U6	median (20.99)	I-IV	60	OS	3.17 (2.39, 4.179)	Reported	Age, Gender, WHO grade, KPS	8
Zh^25^	China	(Asian)	124	tissue	qRT-PCR	Has-miR-16	median (—)	I-IV	98	OS	1.882 (1.07, 3.308)	Reported	Gender, Age, WHO grade, hsa-miR-106a, hsa-miR-181b	9
Ilhan-Mutlu^31^	Austria	(Non-Asian)	15	tissue	qRT-PCR	RNU6B	median (—)	IV	44	PFS	1.23 (0.41, 3.72)	Data-extrapolation	—	5

*The cut-off values to define the high- or low-expression of miR-21.

### Correlation between miR-21 expression and OS

**Figure 2:**
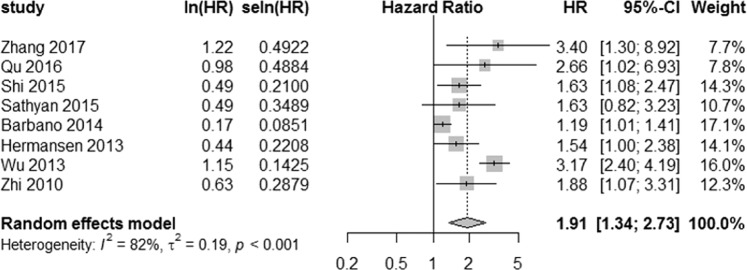
The association between miR-21 expression and OS in eight studies.

Among the nine studies enrolled in evaluating the association of miR-21 expression and glioma prognosis, eight studies provided data on OS, and one study provided data on PFS^31^. For the eight studies with OS data, the random-effect model was adopted to calculated the pooled HR and 95% CI because of the high heterogeneity among studies (*I*^2^ = 82.0%, *P* < 0.001). The pooled result (HR = 1.91, 95% CI: 1.34, 2.73) showed that a higher miR-21 expression significantly predicted a poorer OS in the patients with gliomas (Fig. 2).

### Subgroup analyses

Subgroup analyses were conducted to explore the causes of heterogeneity according to locations of the sample collection, methods of the miRNA assay, cut-off values of miR-21 expression, follow-up periods, and adjustment variables (Table 2). The subgroup analysis by location of sample collection showed that a higher expression of miR-21 predicted poorer prognoses in Asian glioma patients (HR = 2.37, 95% CI: 1.68, 3.35) compared with that in Non-Asian patients (HR = 1.25, 95% CI: 1.07, 1.45). The subgroup analysis by different miRNA test methods indicated that the qRT-PCR group (HR = 1.99, 95% CI: 1.31, 3.01) predicted worse prognoses with higher expressions of miR-21 in the glioma patients. Concerning subgroups by different cut-off values, a significant HR was found in the median group (pooled HR = 2.09, 95% CI: 1.41, 3.11), while the mean group showed an insignificant pooled HR (HR = 1.51, 95% CI: 1.00, 2.27). According to the subgroups of follow-up time, a follow-up time of > 60 months group showed a significant HR (pooled HR = 1.36, 95% CI: 1.19, 1.56), indicating that higher miR-21 expression was associated with a poorer OS in glioma patients. The subgroup analysis by the adjustment variables of the multivariate Cox regression analysis showed that higher expression of miR-21 predicted poorer prognoses (HR = 1.78, 95% CI: 1.15, 2.75).

**Table 2:** Subgroup analyses of associations between miR-21 expression and OS.

Subgroup	Number of Studies	Number of Patients	Pooled model	Pooled HR (95% CI)	Heterogeneity		Publication bias (*P*-value)	
					*I*^2^ (%)	*P*-value	Begg’s test	Egger’s test
All	8	1059	random	1.91 (1.34, 2.73)	82	<0.001	0.216	0.236
**Locations**								
Asian	5	509	random	2.37 (1.68, 3.35)	52	0.083	0.624	0.719
Non-Asian	3	640	fixed	1.25 (1.07, 1.45)	0	0.400	0.602	0.164
**Method**								
qRT-PCR	7	960	random	1.99 (1.31, 3.01)	85	<0.001	0.293	0.262
ISH	1	189	—	1.55 (1.00, 2.38)	—	—	—	—
**Cut-off value**								
Median	4	705	random	2.09 (1.41, 3.11)	67	0.028	0.497	0.195
Mean	3	409	random	1.51 (1.00, 2.27)	63	0.068	0.117	0.087
1.5-fold	1	35	—	2.66 (1.02, 6.93)	—	—	—	—
**Follow-up (month)**								
≤60	1	152	—	3.17 (2.40, 4.19)	—	—	—	—
>60	7	997	fixed	1.36 (1.19, 1.56)	43	0.107	0.051	0.000
**Adjustment variables**								
Yes	5	848	random	1.78 (1.15, 2.75)	89	<0.001	0.624	0.447
No	3	211	fixed	2.22 (1.37, 3.59)	0	0.433	0.117	0.165

### Sensitivity analysis

A sensitivity analysis showed that the removal of individual studies, in turn, did not change the HR effect of the combined effect (range of pooled HRs: 1.36–2.10, all lower limits of the 95% CIs > 1.0) (Table 3), indicating that the result was stable in the meta-analysis.

**Table 3:** Sensitivity analysis of pooled HRs of higher miR-21 expression for OS.

Study Omitted	Pooled HR	95% CI	Heterogeneity	
			*I*^2^ (%)	*P-*value
Zhang^28^	1.82	(1.26, 2.63)	84	<0.001
Qu^27^	1.86	(1.28, 2.70)	84	<0.001
Shi^23^	1.97	(1.30, 2.99)	85	<0.001
Sathyan^30^	1.95	(1.32, 2.89)	85	<0.001
Barbano^26^	2.10	(1.56, 2.82)	54	0.042
Hermansen^29^	1.99	(1.31, 3.01)	85	<0.001
Wu^24^	1.36	(1.19, 1.56)	43	0.107
Zhi^25^	1.92	(1.29, 2.86)	84	<0.001

### Publication bias

Publication bias was evaluated by Begg’s test and Egger’s test. For the eight studies, the Begg’s test (*P* = 0.216), and Egger’s test (*P* = 0.236) provided no evidence of publication bias (Table 2).

### Correlation between miR-21 expression and PFS

In this meta-analysis, only one study exploring the relationship between miR-21 expression and the survival outcome on PFS in glioma patients was included. No significant HR was reported in the study (HR = 1.23, 95% CI: 0.41, 3.72).

### TCGA data extraction

Of the glioma patients in TCGA dataset, 641 glioma patients were selected according to the selection criteria to verify the prognostic significant of miR-21, and the clinical features of the 641 patients are summarized in Table 4.

**Table 4:** Clinical information of glioma patients from TCGA dataset (n = 641).

Variables	Overall (n = 641)	high miR-21 expression (n = 330)	low miR-21 expression (n = 311)	*P*-value*
Average age at diagnosis (mean ± standard deviation, year)	51.4 ± 15.2	55.1 ± 13.7	47.6 ± 15.7	<0.001
Grade (n, II/III/IV)	110/136/395	23/54/253	87/82/142	<0.001
Gender (n, male/female)	364/277	199/131	165/146	0.064
KPS (n, <80/≥80)	123/518	78/252	45/266	0.003
Median OS time (day)	714	550	1315	<0.001
Median PFS time (day)	463	11.2	32.7	<0.001

*Comparison of high miR-21 expression group and low miR-21 expression group.

### TCGA data validation

**Figure 3:**
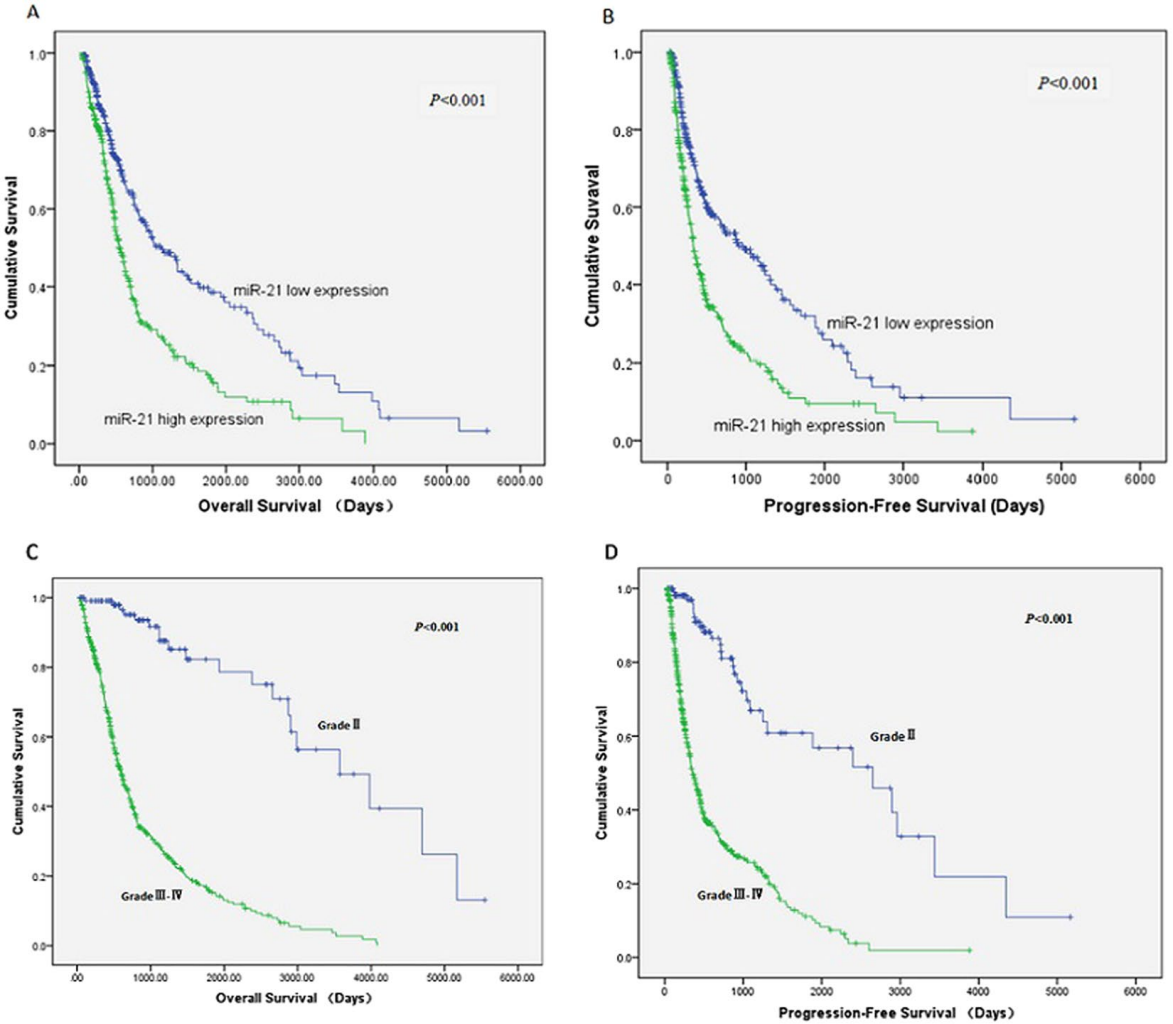
Kaplan-Meier estimates for glioma patients from the TCGA glioma dataset. (**A**) Kaplan-Meier estimates of OS for groups with high and low miR-21 expression; (**B**) Kaplan-Meier estimates of PFS for groups with high and low miR-21 expression; (**C**) Kaplan-Meier estimates of OS for groups with grade II and grade III–IV; (**D**) Kaplan-Meier estimates of PFS for groups with grade II and grade III–IV.

To validate the results of the meta-analysis, the TCGA glioma dataset was used to analyze the relationship between miR-21 expression and survival in the patients with gliomas. Figure 3 shows the Kaplan-Meier estimates for the high miR-21 expression group and the low miR-21 expression group. The results of a log-rank test showed that the glioma patients with high levels of miR-21 expression had a poorer OS (*P* < 0.001) (Fig. 3A) and a poorer PFS (*P* < 0.001) (Fig. 3B), and the results of a log-rank test showed that the glioma patients with grade III–IV had a poorer OS (*P* < 0.001) (Fig. 3C) and a poorer PFS (*P* < 0.001) (Fig. 3D). A multivariate Cox regression analysis indicated that the OS-related variables were miR-21 expression, tumor grade, age at diagnosis, gender, and KPS (Table 5), while the PFS-related variables were miR-21 expression, tumor grade, age at diagnosis and gender (Table 6). Meanwhile, the results of the multivariate Cox regression analysis showed that high miR-21 expression was an independent prognostic biomarker for a poorer OS (HR = 1.27, 95% CI: 1.01, 1.59) and poorer PFS (HR = 1.46, 95% CI: 1.17, 1.82) in patients with gliomas (Tables 5 and 6).

**Table 5:** Multivariate Cox regression analysis for OS in glioma patients.

Variables	HR	95% CI	*P*-value
miR-21 (high/low)	1.27	(1.01, 1.59)	0.042
Grade (III–IV/II)	6.83	(4.06, 11.50)	<0.001
Age at diagnosis (≥50/<50)	2.55	(1.98, 3.29)	<0.001
Gender (male/female)	1.32	(1.06, 1.65)	0.014
KPS (<80/≥80)	2.11	(1.62, 2.75)	<0.001

**Table 6:** Multivariate Cox regression analysis for PFS in glioma patients.

Variables	HR	95% CI	*P*-value
miR-21 (high/low)	1.46	(1.17, 1.82)	0.001
Grade (III–IV/II)	3.96	(2.64, 5.96)	<0.001
Age at diagnosis (≥50/<50)	1.84	(1.46, 2.32)	<0.001
Gender (male/female)	1.29	(1.04, 1.60)	0.023
KPS (<80/≥80)	0.97	(0.71, 1.31)	0.828

## Discussion

Alterations in the expression levels of specific miRNAs can be easily and stably detected in tumor tissues^17–20^, plasma and serum^36,37^ and cerebrospinal fluid (CSF)^38,39^. Therefore, miRNAs are potential tumor biomarkers^40–43^. In recent years, an increasing number of studies have shown that miRNAs are valuable for predicting tumor prognosis^20,22,40–43^. Previous studies have shown that the role of miR-21 in predicting the prognoses of glioma patients remains controversial, possibly because small sample sizes could have led to an inadequate statistical ability to detect certain relationships in individual studies. Therefore, our findings in this meta-analysis and the multivariate Cox regression analysis of the TCGA glioma data on miR-21 expression to assess the prognostic value of miR-21 in glioma patients are more believable.

A meta-analysis to assess the relationship between miR-21 expression and OS in glioma patients was first performed in 2016^35^. Compared with that study, our current meta-analysis included four new eligible studies, and we also analyzed the relationship between miR-21 expression and PFS in glioma patients. In our study, we found that the expression level of miR-21 is associated with prognosis, with a pooled HR of 1.91 (95% CI: 1.34, 2.73). This suggests patients with higher miR-21 expression have shorter OS.

Gliomas are clinically complex tumors with various manifestations. There are many factors affecting the prognoses of glioma patients. In analyzing the relationship between miR-21 and prognosis, it is important to consider confounding effects caused by the degree of malignancy as well as other factors^23–31^. In this meta-analysis, five included studies used a multivariate Cox regression analysis to study the relationship between miR-21 expression and OS, and in each of these five studies, the covariate confounding effect was adjusted by performing a multivariate Cox regression analysis. The pooled HR for the five included articles was 1.78 (95% CI: 1.15, 2.75), indicating that glioma patients with higher miR-21 expression have shorter OS.

In addition, the multivariate Cox regression analysis of covariates (the miR-21 expression, grade, age at diagnosis, gender, and KPS) in the TCGA glioma datasets revealed that overexpression of miR-21 is a potential independent prognostic biomarker of poorer OS (HR = 1.29, 95% CI: 1.01, 1.59) and poorer PFS (HR = 1.46, 95% CI: 1.17, 1.82) in glioma patients. Therefore, based on the meta-analysis results and the validation results by the TCGA dataset, we believe that miR-21 is a significant and independent prognostic biomarker for a glioma patient survival.

The associations between elevated miR-21 expression and poor survival can partly be explained by its role in the cascade of tumorigenesis and progression. The gene miR-21 is up-regulated in gliomas, and its oncogenic effect may be mediated through regulation of certain transcriptional targets and downstream signaling pathways. Currently, some tumor suppressor genes have been identified as targets of miR-21, such as programmed cell death 4 (PDCD4)^44,45^ and phosphatase and tensin homolog (PTEN)^46^. Furthermore, cellular pathways such as p53 and the PI3K-Akt pathway, are also part of the miR-21 regulatory network^47,48^. By attenuating or inhibiting these tumor suppressor genes, miR-21 can promote tumor proliferation, invasion, and metastasis, and reduce sensitivity to chemotherapy, thereby affecting the prognosis of glioma patients.

Because of the existence of heterogeneity among included studies, subgroup analyses were conducted according to locations of sample collection, methods of the miRNA assays, cut-off values of miR-21 expression, and follow-up periods. The results showed that a higher expression of miR-21 was predictive of poorer prognoses in Asian glioma patients (HR = 2.37, 95% CI: 1.68, 3.35); and the prognostic effect of miR-21 in the glioma patients could be influenced by the follow-up periods, test methods, and cut-off values of miR-21 expression. The different adopted therapeutic regimen for the glioma patients and the different reference of miR-21 quantified in the tumor tissues may lead to heterogeneity between the included studies. There were three included studies conducted in non-Asian subjects, one of which^29^ employed an ISH technique and the other two of which^26,30^ employed qRT-PCR technique. Although the methodological approach to quantify miR-21 were different, the heterogeneity among the three studies was not significant (*I*^2^ = 0%, *P* = 0.400). In the included studies, the different cut-off values were used to define the high- or low-expression of miR-21, which may affect the power of miR-21 as a prognostic biomarker in glioma. The random effect model and subgroup analyses conducted according to the cut-off values of miR-21 expression were performed to weaken the influence of heterogeneity on the conclusion of miR-21 as a predictive biomarker in glioma. There was no significant publication bias identified in this meta-analysis, and the result of the sensitivity analyses also showed the robustness of our results.

The literature review for this updated meta-analysis was thorough and adequately chosen. Concurrently, the TCGA data used to verify the meta-analysis results of this study are standardized data, and the sample size of the TCGA data set is large, so the results of this study are reliable. However, it should be noted that there were some limitations existed in our study. Firstly, there was heterogeneity in the included studies. Although we performed subgroup meta-analyses and adopted a random-effect model to minimize the effects of the heterogeneity, the heterogeneity among the studies may still affect the reliability of the combined results. For example, in a subgroup analysis of different miR-21 detection methods, the expression of miR-21 was verified by qRT-PCR in seven studies, and heterogeneity existed in these seven studies (*I*^2^ = 85%, *P* < 0.001). A possible reason is that the subjects came from different countries (Asian and non-Asian), and the tumor grades varied by study (IV, III–IV, II–IV, I–IV). Secondly, the cut-off values to define the high- or low-expression of miR-21 were different in the included studies. Thirdly, a few studies only provided results calculated by a univariate analysis, which did not adjust for the impacts of certain variables on the prognosis, such as patients’ age, gender, treatment received, and tumor grade. Therefore, the authentic prognostic value of miR-21 may be affected in the glioma patients. Fourthly, because there were relatively few original studies that met the inclusion criteria, the total sample size of this meta-analysis (n = 1,059) is small, which may reduce the statistical power of the summarizing results. Fifthly, most of the included studies were based on Chinese patients, while only three included studies were conducted in non-Asian subjects. Sixthly, there may be some possible confounding parameters influencing the prognostic role of miR-21, while the confounding effects of the clinical features were only considered in this study of the relationship between miR-21 and the prognosis of glioma patients. Considering the limitations of the included studies in this meta-analysis, we will continue to focus on relevant studies and improve the limitations of this study to get a more reliable conclusion.

In summary, elevated miR-21 expressions could indeed predict worse OS in glioma patients, and the prognostic effect of miR-21 was more prominent in the Asian group. There are still some detailed issues that need to be addressed for the clinical application of miR-21 as a prognostic marker in gliomas. For examples, it is necessary to establish a standard cut-off value defining the high and low levels of miR-21 expression, and a standard method for detecting the expression of miR-21 to ensure accuracy and comparability in glioma patients. We will track the research reports of the prognostic value of miR-21 in gliomas, and explore the influence of the difference between the detection of miR-21 in tumor tissue and liquid biopsy on the prognostic value of miR-21 in gliomas.

## Material and Methods

### Meta-analysis

#### Search strategy

We conducted a systematic search for available literature in the electronic databases Embase, PubMed, Web of Science, Chinese national knowledge infrastructure (CNKI), China biomedical literature service system (SinoMed), and Wanfang up to May 2018. The search terms used were “miR-21 OR microRNA-21”, “glioma OR glioblastoma OR GBM OR astrocytoma”, and “prognosis OR prognostic OR survival OR recurrence”. These three search terms were combined by the Boolean operator “AND”. In addition, we sought eligible studies by conducting a manual search of references from relevant articles and reviews to avoid missing potentially related articles.

### Inclusion criteria

Two investigators independently determined the eligibility for each included article in the meta-analysis according to the inclusion criteria and exclusion criteria. Any disagreements during the selection process were resolved by discussion with a third author.

Eligible studies: (1) described the correlation between miR-21 expression and survival in glioma patients; (2) provided HRs with 95% CI directly, or key information to calculate HR indirectly, such as K-M curves and original survival data; (3) described a case-control study or cohort study; (4) categorized glioma patients into low- and high-expression groups only based on the miR-21 expression; and (5) were written in English or Chinese.

The exclusion criteria were as follows: (1) reviews, basic laboratory research, and conference abstracts; and (2) duplicated or overlapped studies.

### Quality assessment

The quality of all included studies was assessed using the NOS^49^. The NOS contains eight items that are categorized into three groups (selection, comparability, and outcome or exposure). A star system is employed to assess the quality of the included study, so that the highest quality study is assigned to a maximum of one star per item, except for the item related to the comparability that allows for two stars. NOS ranges from zero to nine stars, and the highest quality study was assigned nine stars. Each included study with more than five stars was considered to be high quality^50^.

### Data extraction

For each included study, the following data were extracted: (1) first author’s name, publication year, and location of sample collection; (2) characteristics of the studied population (sample size, follow-up period, tumor grade, sampling type); (3) miRNA test method, cut-off value to define high- or low-expression of miRNA, extracting method of HRs (95% CIs), outcome (OS or PFS), and NOS; and (4) HRs of miR-21 expression associated with survival of glioma patients in terms of OS and PFS with 95% CIs. If without HRs (95% CIs), and only the raw data were provided in the study, the HRs (95% CIs) were calculated from the raw data; or, if without HRs (95% CIs), and only the K-M curves were provided in the study, the HRs (95% CIs) were extracted using the method provided by Tierney and Parmar^51,52^.

### Quantitative data synthesis

HR with 95% CI was used to evaluate the effect size for the OS or PFS. HRs from individual studies were transformed to their logarithms to stabilize the variances and normalize the distributions. To assess the heterogeneity of HRs across the included studies, the Cochran Q’s statistic and Higgins *I*^2^ statistic were calculated. A fixed-effect model (Mantel-Haenszel method) was adopted to calculate the pooled HRs (95% CIs) if the heterogeneity was absent among studies (*P* > 0.10 and *I*^2^ < 50%), while if the heterogeneity was observed among studies, the random-effect model (DerSimonian-Laird method) was selected to calculate the pooled HRs (95% CIs), and the subgroup meta-analyses were performed (*P* ≤ 0.10 or *I*^2^ ≥ 50%). A sensitivity analysis was used to evaluate the stability and reliability of the results, by omitting one individual study at a time and analyzing the remaining studies to detect whether the results were influenced excessively by any single study.

Begg’s test and Egger’s test were both used to test the significance of publication bias, with a *P*-value ≤ 0.10 considered significant. All *P*-values were two-sided. All calculations were carried out using R (version 3.2.3, R Foundation for Statistical Computing, Vienna, Austria).

### TCGA dataset analysis

The miR-21 expression data and the clinical data of the LGG patients and the GBM patients were obtained from the TCGA database. MiR-21 expression was measured by Illumina Hi-Seq platform in the LGG dataset and Agilent 8 × 15 K Human microRNA platform in the GBM dataset. The inclusion criteria for the patients in the LGG and GBM datasets were as follows: (a) miR-21 expression level data and the corresponding follow-up data were available; (b) the OS or PFS were ≥ 30 days; and (c) clinical data of the patients, such as age at diagnosis, gender, tumor grade, and KPS, were available. The high- and low-expression groups were distinguished by the median value of the miR-21 expressions. Unpaired *t* test and chi-square test were used for the comparison of high miR-21 expression group and low miR-21 expression group. The survival curves were estimated by the Kaplan–Meier method. Survival differences between the high-expression group and the low-expression group were assessed by a log-rank test. A multivariate Cox regression analysis was used to identify miR-21 expression as an independent prognostic biomarker. All the statistical analyses were performed using PASW Statistics Version 18.0 (SPSS Inc., Chicago, Illinois, USA).
